# Association between life's essential 8 and Parkinson's disease: a case–control study

**DOI:** 10.1186/s12889-025-21648-0

**Published:** 2025-02-01

**Authors:** Jiaxin Fan, Yanfeng Wang, Xingzhi Guo, Shuai Cao, Shuqin Zhan, Rui Li

**Affiliations:** 1 Department of Geriatric Neurology, Shaanxi Provincial People’s Hospital, No. 256 West Youyi Road, Xi’an, 710068 China; 2 Shaanxi Provincial Clinical Research Center for Geriatric Medicine, Xi’an, China; 3 Department of Neurology, The Second Affiliated Hospital of Xi’an Jiaotong University, No. 157 West Five Road, Xi’an, 710004 China; 4 Department of Oncology Surgery, Shaanxi Provincial People’s Hospital, Xi’an, China; 5 Department of Clinical Laboratory, Affiliated Hospital of Yan’an University, Yan’an, China; 6 Institute of Medical Research, Northwestern Polytechnical University, Xi’an, China; 7 Department of Orthopedics, Civil Aviation General Hospital, Beijing, China

**Keywords:** Life's essential 8, Cardiovascular health, Parkinson's disease, NHANES, Beneficial insight

## Abstract

**Objectives:**

Life's essential 8 (LE8) is an emerging approach for accessing and quantifying cardiovascular health (CVH), but the effect on Parkinson's disease (PD) is still unclear. This study aimed to elucidate the association between LE8 metrics and PD in the US adults.

**Methods:**

Data of 26,975 participants were extracted from the last 7 National Health and Nutrition Examination Survey (NHANES) cycles (2005–2018). The LE8 metrics were calculated according to the American Heart Association criterion, and participants were divided into 3 groups using tertile range. Multivariate logistic regression models were constructed to explore the association between LE8 metrics and PD. Sensitivity analysis was conducted to verify robustness. A nonlinear linkage was evaluated via restricted cubic spline (RCS). The stability of this effect was validated by subgroup analysis and interaction test.

**Results:**

A total of 26,975 eligible participants (including 271 PD cases and 26,704 non-PD cases) were included in this study. The multivariate logistic regression models revealed a reverse association of continuous LE8 metrics with PD with ORs of 0.97 (unadjusted model [95% CI: 0.96–0.98, *P* < 0.01], partially adjusted model [95% CI: 0.97–0.98, *P* < 0.01], fully adjusted model [95% CI: 0.95–0.98, *P* < 0.01]). Compared to those of low group, the ORs for high group were 0.37 (95% CI: 0.27–0.50, *P* < 0.01) in unadjusted model, 0.51 (95% CI: 0.36–0.72, *P* < 0.01) in partially adjusted model, and 0.51 (95% CI: 0.32–0.81, *P* < 0.01) in fully adjusted model. The sensitivity analysis ensured the robustness of the observed LE8-PD association. A nonlinear relationship (*P*_nonlinearity_ < 0.01) was observed via RCS analysis. The subgroup analysis showed that participants'gender might impact the strength of LE8 metrics-PD association (*P*_interaction_ = 0.029).

**Conclusions:**

CVH, as delineated by LE8 metrics, was reversely associated with PD in the dose–response pattern, more pronounced in female compared to male. These findings highlight the potential of the LE8 metrics to guide targeted strategies for addressing gender-based CVH disparities, offering beneficial insights for the tertiary prevention of PD.

**Supplementary Information:**

The online version contains supplementary material available at 10.1186/s12889-025-21648-0.

## Introduction

Parkinson's disease (PD) is the second frequent neurodegenerative condition, presenting a slow deterioration with functional independence and quality of life for affected individuals [1, 2]. The number of global PD patients has reached about 6.1 million in 2016, which is projected to increase to 10 million by 2030 [3, 4]. The incidence of PD patients increases 5–10 folds in the sixth to the ninth decades of life course, and the ageing of the global population makes this trend even more pronounced [5]. Along with this increase, the familial and societal economic burdens of PD have also escalated. In addition to classical motor symptoms such as bradykinesia, rigidity with cogwheeling, rest tremor, and postural abnormality, many patients develop a succession of nonmotor symptoms, including sleep disturbances, neuropsychiatric disorders, and cognitive impairment. Nonmotor symptoms can antedate or delay the onset of motor symptoms, increasing in severity with disease duration. Dopaminergic drugs and functional neurosurgery remain the two cornerstones of PD treatment. However, these approaches neglect the conspicuous heterogeneity of afflicted individuals and cannot arrest the progression of PD. Considering that genetic, environmental, and lifestyle dysfunctions are the etiologies of PD, a promising shift from a focus solely on disease treatment to intervening the modifiable risk factors (including sleep, diet, and physical activity) is urgently needed for burden-relieving potential.

During the search to investigate the risk factors of PD, a vast array of studies supports that there are several common factors between PD and cardiovascular disease (CVD), which may be attributed to the common pathogenesis of both diseases, such as oxidative stress, inflammation, and lipid metabolism [6]. However, whether treating these shared cardiovascular risk factors affects the occurrence and progression of PD remains controversial. As a quantification of CVD, cardiovascular health (CVH) was first established and anchored on 7 health components in 2010. The ideal CVH consists of a well-balanced diet, physical activity, no smoking, normal body mass index (BMI), normal glucose, normal total cholesterol, and normal blood pressure, and is also known as “Life's Simple 7” (LS7) [7]. Over time some indicators of LS7 may not provide a comprehensive appreciation of health behaviors and clinical practices in the contemporary environment, especially dietary components [8]. In addition, subsequent evidence highlights that sleep health also contributes to risks for health outcomes [9, 10]. To address those limitations of LS7, the American Heart Association (AHA) proclaimed an improved algorithm——“Life's essential 8” (LE8) to measure and prompt CVH, which is a powerful health trajectory system with enhanced sensitivity to interindividual and intraindividual changes over time [8]. When all components of LE8 (including added sleep health and updated 7 parts of LS7) are at ideal levels, individuals will be more inclined to obtain favorable effects on CVH promotion. The optimal CVH is not only linked to lower CVD mortality, greater longevity, and better quality of life, but also associated with lower risks of non-CVD diseases, such as cancer, chronic obstructive pulmonary disease, and dementia [11, 12]. To date, much of the existing research has focused on separate effect of each part of the LE8 on PD, and there is a paucity of evidence objectively assessing the association of the LE8 with PD. The application of LE8 metrics enables a more precise evaluation of individual PD changes from a holistic perspective, we thus assume that the integrated effect conferred by optimal CVH on preventing PD may be greater than the sum of its parts.

In the present study, from the perspective of newly published LE8 metrics as an evaluation of CVH, we investigated the association between LE8 metrics and PD in nationally representative data of United States (US) adults.

## Methods

### Study strategy and participants

**Fig. 1:**
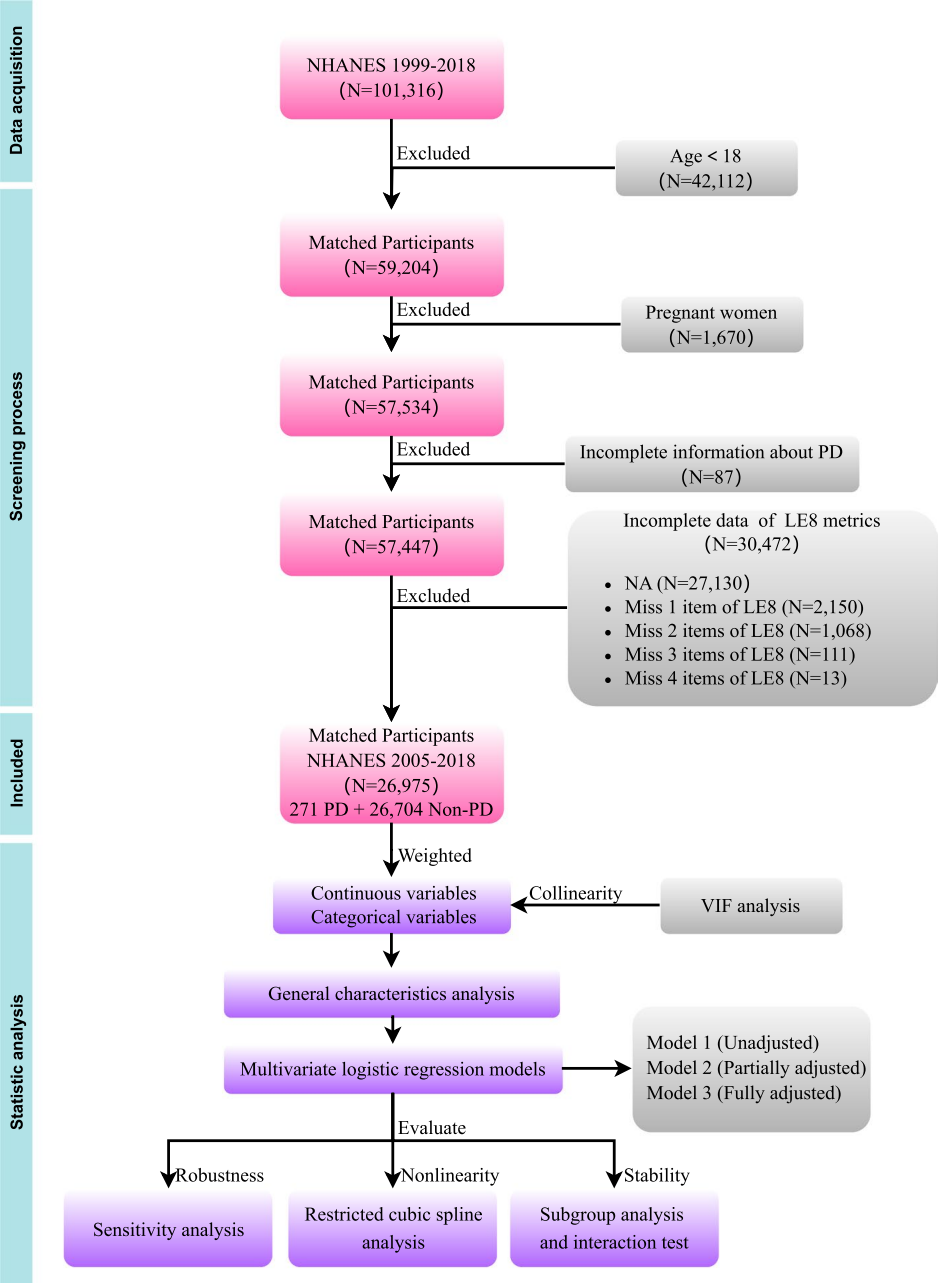
Study strategy and screening process of this study. NHANES, National Health and Nutrition Examination Survey; PD, Parkinson's disease; LE8, Life's essential 8; VIF, Variance Inflation Factor

As a comprehensive, flexible, stratified sampling program on the US population' health and nutritional status, National Health and Nutrition Examination Survey (NHANES) data had been collected and released biennially without disruption since 1999 [13]. The trained staff gathered dietary, socioeconomic, demographic, anthropometric, laboratorial, and clinical information via face-to-face household interviews and mobile examination centers. Due to the COVID-19 pandemic, the NHANES suffered from > 1 year gap in data collection [14]. And sleep health data in LE8 metrics were initially collected in 2005 [15]. Thus, we actually used publicly available NHANES data from 2005 to 2018, spanning 7 cycles. Among the 101,316 participants recruited in NHANES 1999–2018, 42,112 participants who were < 18 years old were excluded firstly, 1,670 pregnant women were excluded. Next, 87 participants who did not know if they had been diagnosed with PD. Then, 30,472 participants with incomplete data of LE8 metrics were excluded, specifically, 2,150 participants were missing 1 item, 1,068 participants were missing 2 items, 111 participants were missing 3 items, 13 participants were missing 4 items, and 27,130 participants were completely missing 8 items. Ultimately, 26,975 eligible participants (including 271 PD cases and 26,704 non-PD cases) were included. The NCHS Ethics Review Board had approved this survey and the informed consents of all participants were signed. Considering the attributes of open access and deidentification, this study was exempted from the ethical approval of the Shaanxi Provincial People's Hospital Ethics Committee. The screening process and study strategy were showed in Fig. 1.

### Assessment of PD

Following previous methodology, participants were assessed PD depended on the use of specific medications to treat PD in NHANES database, which was proven to be the best available approach for identifying PD cases [16]. In this sense, once a participant was reported to actively take any secure anti-PD medication, such as levodopa, methyldopa, carbidopa, entacapone, or pramipexole, he/she was considered to have PD [17]. Conversely, if a participant was not reported to use any of the aforementioned medications, he/she was categorized as not having PD.

### Ascertainment of covariates

The continuous covariates consisted of age, physical activity (PA), and laboratory-reported indicators, such as total cholesterol (TC), high-density lipoprotein (HDL), glycated hemoglobin (HbA1c), blood urea nitrogen (BUN), alanine transaminase (ALT), and aspartate aminotransferase (AST). The categorical covariates included: 1) Gender was classified as female and male; 2) Ethnicity was divided into 4 groups: Mexican American, Non-Hispanic Black, Non-Hispanic White, and Other Race; 3) Educational level was categorized into < High School, High School, > High School; 4) Poverty-to-income ratio (PIR) was applied to evaluate socioeconomic condition, which was divided into < 1.3, 1.3 ≤ PIR < 3.5, and ≥ 3.5; 5) Marital status was categorized into unpartnered and partnered; 6) BMI was divided into < 24 kg/m^2^ and ≥ 24 kg/m^2^; 7) Alcohol drinking was categorized into 5 strata: none, former, light, moderate, and heavy; 8) Smoking status was divided into never, former, and current; 9) Participants'medical history pertaining to CVD, diabetes mellitus (DM), hypertension, and stroke were diagnosed through related-drug treatment, self-reporting, combined with index measurements.

### Measurement of LE8 metrics

**Fig. 2:**
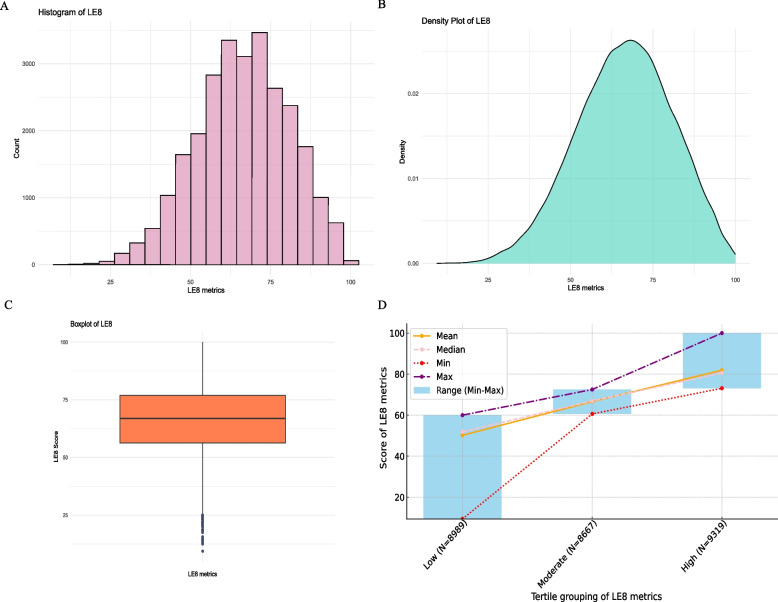
The distribution and grouping of LE8 metrics. The histogram of LE8 metrics (**A**). The density plot of LE8 metrics (**B**). The boxplot of LE8 metrics (**C**). The details of LE8 metrics based on tertile grouping (**D**). LE8, Life's essential 8

The LE8 metrics, a novel tool for gauging CVH, were a combination of 4 health behaviors and 4 health factors. The thorough scoring algorithm of each LE8 component was consistent with previous methods [18, 19]. The integrated LE8 metrics were calculated relied on the unweighted average of each component's score (0–100 scale). We analyzed the continuous LE8 metrics adherence to tertile ranges through the following steps: (1) Sorting: Data from 26,975 eligible participants were arranged in ascending order based on LE8 metrics, and the distribution of LE8 metrics was presented using a histogram (Fig. 2A), density plot (Fig. 2B), and boxplot (Fig. 2C). The LE8 metrics followed a nearly normal distribution, ranging from 0 to 100, with no extreme values observed. (2) Defining cut-off points: Tertile thresholds were determined, with the first tertile corresponding to the 33.3rd percentile of LE8 metrics and the second tertile to the 66.7th percentile. (3) Grouping: The data were divided into three groups based on the thresholds: low (0 < LE8 ≤ 60.0), moderate (60.0 < LE8 ≤ 73.0), and high (73.0 < LE8 ≤ 100.0), using the low group as the reference. Figure 2D presented the details of LE8 metrics grouping. For the 4 health behaviors, data on sleep patterns, nicotine exposure, and physical activity were derived from standardized questionnaires, while dietary data were determined by alternative Healthy Eating Index 2015 base on two 24-h dietary recalls [20]. Information on BMI, blood glucose, blood pressure, and non-HDL cholesterol levels among the 4 health factors were procured laboratory and physical examinations at specialized mobile centers complying with established protocols and computational formulas.

### Statistical analysis

Using R software (4.2.3 version) and EmpowerStats (4.0 version) for Windows, we carried out the graphics and analyzed the data. Following the authoritative recommendations from the NCHS [21], the appropriate sample weights were conducted to account for the oversampling, nonresponse, sampling error, and poststratification, making the estimates more nationally representative of the US adults [22]. Briefly, sampling weights were calculated based on adjusted response rates and selection probabilities. Specifically, data from NHANES 2005–2018 (7 cycles) were weighted using the 7-period Full Sample 2-Year MEC Exam Weight (WTMEC2YR), with the adjusted weight calculated as WTMEC2YR × 1/7. To reflect the survey's complex design, the analysis incorporated strata (SDMVSTRA) and clusters (SDMVPSU), improving the precision and reliability of variance estimates. These techniques were implemented to minimize variability and ensure unbiased results. The weighted mean ± standard error (SE) was used to outline the continuous variables, and the weighted percentage (%) ± SE was used to outline the categorical variables. Differences among the low, moderate, and high groups were assessed by weighted ANOVA and weighted chi-square test. The Variance Inflation Factor (VIF) analysis was used to evaluate the collinearity among variables.

To address missing values in our variables, we utilized the "mice" package in R to conduct Multiple Imputations (MIs), a well-established rule-of-thumb method for handling missing data [23]. The process involved the following steps: (1) Identify missing data patterns: We calculated the missing rates for all variables and visualized the distribution of missing values using bar plots (Figure S1). This facilitated interpretation of the missingness patterns, such as Missing at Random (MAR) or Not Missing at Random (NMAR). (2) Generate imputed datasets: Using predictive mean matching (pmm), we handled the missing values by generating 10 separate imputed datasets (dataset 1–10) with the Multiple Imputation by Chained Equations (MICE) function. Each dataset imputed missing values through predictive models that leverage the relationships among observed variables. (3) Fit statistical models on each dataset: Statistical models were fitted individually to each imputed dataset. We conducted rigorous sensitivity analyses to evaluate the robustness of the findings and to validate the effectiveness of the imputation approach in addressing missing data. (4) Synthesize results across datasets: The results obtained from the 10 imputed datasets were synthesized into comprehensive estimates and confidence intervals. This step accounted for the variability introduced by the imputation process, ensuring statistically robust and credible conclusions [23, 24]. Table S1 showed the overview of missing values of each variable. And Table S2 presented the missing values distribution by LE8 tertile grouping. Table S3 displayed the descriptive statistics of each variable before and after MIs. Following a previously reported threshold screening method [25], we retained variables with missing rates < 10%. Consequently, the PA variable, with a missing rate exceeding 10%, was removed from subsequent analyses.

The association between LE8 metrics and PD were examined using multivariate logistic regression models, and 2 adjusted models were performed to evaluate the sensitivity and robustness of this effect. Specifically, no adjustment was made for the covariates in Model 1, while Model 2 (partially adjusted model) was augmented model by adjusting for age, gender, ethnicity, education level, marital status and PIR. Model 3 (fully adjusted model) further adjusted for BMI, ALT, AST, HbA1c, BUN, HDL, TC, smoking status, alcohol drinking, CVD, DM, stroke, and hypertension on the basis of Model 2, strengthening the adjustment of confounding covariates. Through the above constructed models, we fetched odds ratio (OR) and 95% confidence interval (95% CI) across the whole LE8 metrics groups. Moreover, we also calculated the ORs for each of the 10 imputed datasets and combined them into an overall estimate. To evaluate the potential impact of PA removal on the association of LE8 metrics and PD, we performed a similar reanalysis on the datasets without PA variable. These results were visualized using a forest plot to facilitate comparison and interpretation.

Restricted cubic spline (RCS) plots with 4 knots were constructed to explore the potential nonlinear relationship between LE8 metrics and PD, using the "rms" package in R. The knots were automatically positioned at the 5th, 35th, 65th, and 95th percentiles of the independent variable, LE8. By selecting 4 knots, the fitted model in this study effectively balanced the risks of underfitting and overfitting, providing reliable and interpretable results. Additionally, subgroup analysis was stratified by covariates vulnerable to demographic discrepancies, which was used to assess the stability of the findings. An interaction test was also performed, with the *P* value for interaction used to quantify whether there was a significant interaction effect between two or more independent variables, using forest plot to visualize this process. Statistical significance was denoted at *P* < 0.05.

## Results

### General characteristics of participants

A total of 26,975 participants were eligible in this study. From Table S1 and Figure S1A, we calculated the missing rate of each variable: 31.54% for PA, 7.73% for PIR, 6.75% for alcohol drinking, 0.65% for AST, 0.60% for ALT, 0.36% for BUN, 0.11% for stroke, 0.07% for HbA1c, 0.07% for education level, and 0.01% for CVD. Table S2 and Figure S1B presented the missing data distribution by LE8 tertile grouping. Table 1 listed the general characteristics of 26,975 participants by LE8 metrics category. Compared with those in low and moderate groups, the participants in high group were younger, more likely to be female, and Non-Hispanic White. Participants with higher LE8 metrics had more remarkable family income, greater educational achievement, and more stable marital status. Participants with higher LE8 metrics had lower physical activity levels, and they were less prone to being overweight, with a BMI typically < 24 kg/m^2^. In comparisons of laboratory indicators, LE8 metrics were negatively correlated with TC, HbA1c, BUN, ALT, and AST, whereas positively correlated with HDL. In addition, the participants with higher LE8 metrics were more inclined to be former or light drinkers or never smokers. They presented lower incidence rates of CVD, DM, hypertension, stroke, and PD. The PA variable, with a missing rate exceeding 10%, was removed in accordance with the established threshold protocol in the subsequent analyses [25]. Notably, this removal did not affect the total sample size of 26,975 participants. We also compared the general characteristics of participants with and without PA data (Table S4). The participants without PA data had higher overall LE8 levels and were more likely to fall into the middle and high tertiles of LE8 scores compared to those with PA data.

**Table 1:** General characteristics of the study participants in NHANES 2005–2018, weighted

Characteristics	LE8 metrics			*P*
	**Low** **(0 < LE8 ≤ 60.0)**	**Moderate** **(60.0 < LE8 ≤ 73.0)**	**High** **(73.0 < LE8 ≤ 100.0)**	
**N**	**8,989**	**8,667**	**9,319**	
**Age**, years	52.87 ± 0.24	49.00 ± 0.30	43.65 ± 0.34	< 0.001
**Gender**				< 0.001
Female	50.41 ± 0.76	48.09 ± 0.61	55.83 ± 0.65	
Male	49.59 ± 0.76	51.91 ± 0.61	44.17 ± 0.65	
**Ethnicity**				< 0.001
Mexican American	7.52 ± 0.73	8.60 ± 0.71	7.45 ± 0.60	
Non-Hispanic Black	13.83 ± 0.92	10.75 ± 0.71	7.06 ± 0.51	
Non-Hispanic White	68.57 ± 1.48	69.59 ± 1.35	71.45 ± 1.23	
Other Race	10.08 ± 0.60	11.06 ± 0.60	14.05 ± 0.75	
**Education level**				< 0.001
< High School	6.82 ± 0.45	4.66 ± 0.28	2.94 ± 0.23	
High School	45.39 ± 0.93	35.53 ± 0.85	21.79 ± 0.85	
> High School	47.79 ± 1.02	59.82 ± 0.92	75.26 ± 0.94	
**PIR**				< 0.001
< 1.3	26.13 ± 0.97	19.70 ± 0.67	14.34 ± 0.60	
1.3 ≤ PIR < 3.5	40.89 ± 0.90	36.54 ± 0.92	31.77 ± 0.94	
≥ 3.5	32.99 ± 1.12	43.76 ± 1.09	53.89 ± 1.19	
**Marital status**				< 0.001
Unpartnered	37.97 ± 0.78	35.29 ± 0.82	32.86 ± 0.87	
Partnered	62.03 ± 0.78	64.71 ± 0.82	67.14 ± 0.87	
**BMI**, kg/m^2^				< 0.001
< 24	8.45 ± 0.47	16.65 ± 0.62	39.40 ± 0.73	
≥ 24	91.55 ± 0.47	83.35 ± 0.62	60.60 ± 0.73	
**PA**, MET-h/week	4603.22 ± 169.94	4755.93 ± 120.13	4320.69 ± 97.90	0.002
**TC**, mg/dL	205.32 ± 0.69	196.45 ± 0.75	184.16 ± 0.51	< 0.001
**HDL**, mg/dL	48.31 ± 0.21	52.39 ± 0.23	58.73 ± 0.28	< 0.001
**HbA1c**, %	5.99 ± 0.02	5.54 ± 0.01	5.29 ± 0.01	< 0.001
**BUN**, mg/dL	14.15 ± 0.09	13.74 ± 0.10	13.32 ± 0.09	< 0.001
**ALT**, U/L	27.46 ± 0.33	25.90 ± 0.23	22.64 ± 0.19	< 0.001
**AST**, U/L	26.08 ± 0.23	25.21 ± 0.17	24.48 ± 0.18	< 0.001
**Alcohol drinking**				< 0.001
None	9.45 ± 0.43	10.02 ± 0.50	11.38 ± 0.73	
Former	32.97 ± 0.81	37.29 ± 0.84	42.71 ± 0.90	
Light	15.05 ± 0.58	17.67 ± 0.65	20.03 ± 0.54	
Moderate	21.97 ± 0.60	21.91 ± 0.81	17.05 ± 0.55	
Heavy	20.55 ± 0.71	13.10 ± 0.52	8.83 ± 0.40	
**Smoking status**				< 0.001
Never	33.32 ± 0.75	52.29 ± 0.75	73.39 ± 0.82	
Former	29.44 ± 0.67	27.62 ± 0.70	21.47 ± 0.67	
Current	37.24 ± 0.73	20.09 ± 0.54	5.14 ± 0.33	
**CVD**				< 0.001
No	83.73 ± 0.54	91.82 ± 0.35	96.43 ± 0.25	
Yes	16.27 ± 0.54	8.18 ± 0.35	3.57 ± 0.25	
**DM**				< 0.001
No	76.04 ± 0.58	90.97 ± 0.39	97.86 ± 0.19	
Yes	23.96 ± 0.58	9.03 ± 0.39	2.14 ± 0.19	
**Hypertension**				< 0.001
No	38.79 ± 0.68	59.24 ± 0.68	81.61 ± 0.58	
Yes	61.21 ± 0.68	40.76 ± 0.68	18.39 ± 0.58	
**Stroke**				< 0.001
No	94.39 ± 0.31	97.70 ± 0.19	98.98 ± 0.12	
Yes	5.61 ± 0.31	2.30 ± 0.19	1.02 ± 0.12	
**PD**				< 0.001
No	98.46 ± 0.17	99.04 ± 0.14	99.46 ± 0.10	
Yes	1.54 ± 0.17	0.96 ± 0.14	0.54 ± 0.10	

The continuous variables were described by weighted mean ± standard error (SE), while the categorical variables were described by weighted percentage (%) ± SE. The weighted ANOVA analysis was used to assess the differences of the continuous variables, while the weighted chi-square test was used to assess the differences of the categorical variables

*LE8* Life's essential 8, *PIR* Poverty-to-income ratio, *BMI* Body mass index, *PA* Physical activity, *TC* total cholesterol, *HDL* High-density lipoprotein, *HbA1c* glycated hemoglobin, *BUN* Blood urea nitrogen, *ALT* Alanine transaminase, *AST* Aspartate aminotransferase, *CVD* Cardiovascular diseases, *DM* Diabetes mellitus, *PD* Parkinson's disease

### Association between LE8 metrics and PD

To assess the collinearity among variables, we performed the VIF analysis. According to Table 2, the VIF values ranged from 1.083 (for marital status) to 2.778 (for ALT). Although BMI was one of the components of LE8 metrics, its VIF value was 1.542, while LE8 metrics had a VIF value of 2.717, suggesting no significant collinearity between them. Overall, the VIF values of all variables were all < 5, indicating that the collinearity of these variables was weak and suitable for the next logistic regression analysis. Table 3 presented the association between LE8 metrics and PD. Overall, LE8 metrics were significantly associated with a lower probability of developing PD, as shown across three multivariate logistic regression models, each with an OR of 0.97 (unadjusted model [95% CI: 0.96–0.98, *P* < 0.01], partially adjusted model [95% CI: 0.97–0.98, *P* < 0.01], fully adjusted model [95% CI: 0.95–0.98, *P* < 0.01]). When LE8 metrics were categorized into three groups, we observed a notable decrease in the OR from moderate group (OR: 0.63, 95% CI: 0.48–0.83, *P* < 0.01) to high group (OR: 0.37, 95% CI: 0.27–0.50, *P* < 0.01) in Model 1. After partially adjusting for covariates in Model 2, the ORs of PD were 0.74 (95% CI: 0.55–0.97, *P* < 0.05) in moderate group, and 0.51 (95% CI: 0.36–0.72, *P* < 0.01) in high group compared with low group. This reverse dose–response trend was also observed in Model 3 after adjustment for accessorial covariates, especially in high group (OR: 0.51, 95% CI: 0.32–0.81, *P* < 0.01). These results implied that higher LE8 levels were associated with a reduced likelihood of developing PD.

**Table 2:** The results of VIF to assess collinearity between influencing variables for PD

Variables	VIF	Variables	VIF
Gender	1.228	Age	1.730
Ethnicity	1.095	Education level	1.293
PIR	1.329	Marital status	1.083
BMI	1.542	Smoking status	1.503
Stroke	1.466	Alcohol drinking	1.090
CVD	1.675	HbA1c	1.911
DM	1.959	ALT	2.778
Hypertension	1.504	AST	2.646
BUN	1.270	TC	1.279
HDL	1.378	LE8	2.717

*PIR* Poverty-to-income ratio, *BMI* Body mass index, *TC* Total cholesterol, *HDL* High-density lipoprotein, *HbA1c* Glycated hemoglobin, *BUN* Blood urea nitrogen, *ALT* Alanine transaminase, *AST* Aspartate aminotransferase, *CVD* Cardiovascular diseases, *LE8* Life's essential 8, *DM* Diabetes mellitus

**Table 3:** Multivariate logistic regression models

	**Model 1**^**a**^		**Model 2**^**b**^		**Model 3**^**c**^	
	**OR (95%CI)**	***P***	**OR (95%CI)**	***P***	**OR (95%CI)**	***P***
LE8	0.97 (0.96–0.98)	< 0.001	0.97 (0.97–0.98)	< 0.001	0.97 (0.95–0.98)	< 0.001
**LE8 tertile**						
Low	Reference		Reference		Reference	
Moderate	0.63 (0.48–0.83)	< 0.001	0.74 (0.55–0.97)	0.040	0.79 (0.57–1.10)	0.159
High	0.37 (0.27–0.50)	< 0.001	0.51 (0.36–0.72)	< 0.001	0.51 (0.32–0.81)	0.004

*LE8* Life's essential 8, *OR* Odds ratio, *95%CI* 95% confidence interval

^a^Unadjusted

^b^(Partially adjusted model): Adjusted for age, gender, ethnicity, education level, marital status, PIR

^c^(Fully adjusted model): b + adjusted for BMI, ALT, AST, HbA1c, BUN, HDL, TC, smoking status, alcohol drinking, CVD, DM, stroke, and hypertension

### Sensitivity analysis

**Fig. 3:**
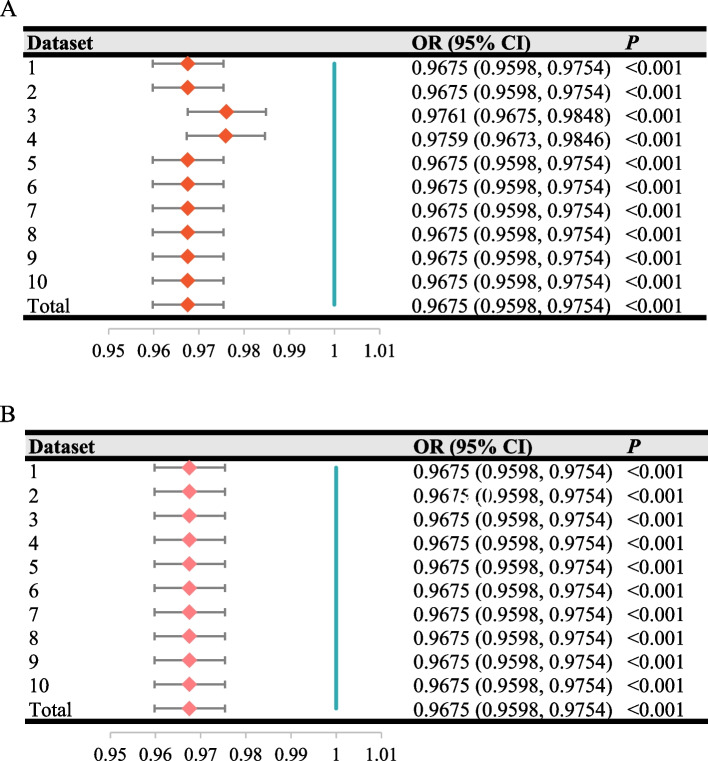
Forest plots from multiple imputations. Forest plot from multiple imputations with PA variable (10 times) (**A**). Forest plot from multiple imputations without PA variable (10 times) (**B**). OR, odds ratio; 95%CI, 95% confidence interval

To evaluate the potential impact of MIs strategies on handling missing values, we generated 10 imputed datasets and 1 combined dataset for sensitivity analysis, which included PA variable. As shown in Fig. 3A, the ORs across all datasets remained consistently close to 0.97, verifying a significant association between higher LE8 levels and a reduced risk of PD. To assess whether the PA removal might introduce underlying bias, we reanalyzed the dataset without PA variable. As illustrated in Fig. 3B, the pooled results across all datasets also remained consistently close to 0.97, matching the ORs obtained from the MIs analysis with PA variable. These findings affirmed that, the MIs strategy and the removal of PA variable were aligned with established best practices and ensured the robustness and reliability of the observed LE8-PD association.

### Restricted cubic spline analysis

**Fig. 4:**
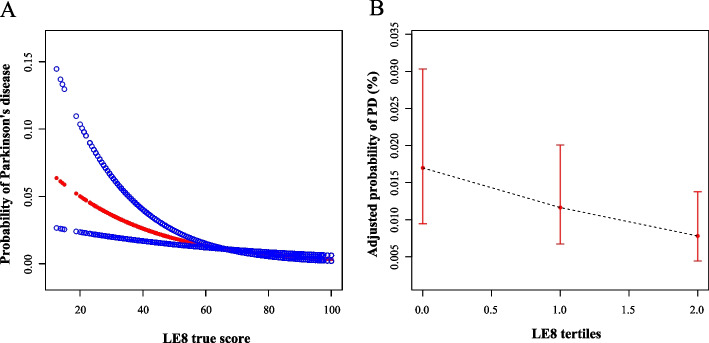
Restricted cubic spline plots with 4 knots. Restricted cubic spline plot illustrating the non-linear association between continuous LE8 true score and the probability of Parkinson's disease. The red line represented the odds ratios, while the blue line denoted the 95% confidence intervals (**A**). Restricted cubic spline plot illustrating the adjusted probability of Parkinson's disease according to LE8 tertiles. Error bars represented 95% confidence intervals (**B**). LE8, Life's essential 8. PD, Parkinson's disease

To evaluate the nonlinear association of LE8 metrics and PD, we conducted RCS plots with 4 knots. As shown in Fig. 4A, the continuous LE8 true score demonstrated a dose–response relationship with PD probability, where higher LE8 scores were associated with a decreased probability of PD (*P*_nonlinearity_ < 0.01). To further examine this nonlinear effect, we categorized LE8 metrics into tertiles. A significant finding from Fig. 4B was that the participants in higher tertiles (higher LE8 scores) tended to had a lower adjusted probability of PD (*P*_nonlinearity_ < 0.01).

### Subgroup analysis and interaction test

**Fig. 5:**
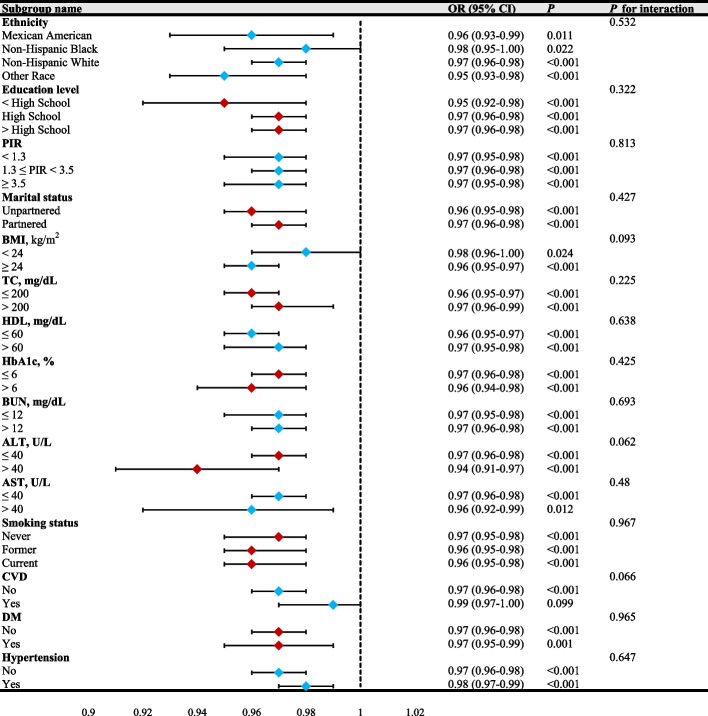
Forest plot subgroup analysis and interaction test between LE8 metrics and PD (*P* for interaction > 0.05). PIR, poverty-to-income ratio; BMI, body mass index; TC, total cholesterol; HDL, high-density lipoprotein; HbA1c, glycated hemoglobin; BUN, blood urea nitrogen; ALT, alanine transaminase; AST, aspartate aminotransferase; CVD, cardiovascular diseases; DM, diabetes mellitus; OR, odds ratio; 95%CI, 95% confidence interval

**Fig. 6:**
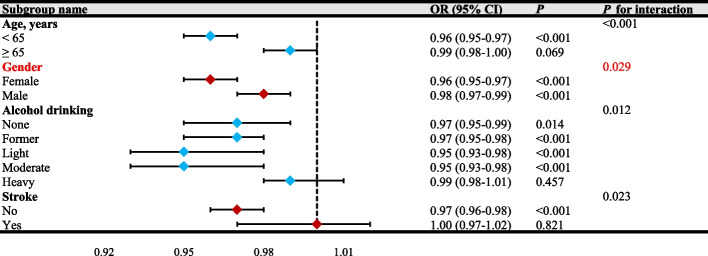
Forest plot subgroup analysis and interaction test between LE8 metrics and PD (*P* for interaction < 0.05). The gender subgroup showed a significant interaction on LE8-PD association, highlighted in red. OR, odds ratio; 95%CI, 95% confidence interval

To investigate the stability of the reverse association between LE8 metrics and PD, we further carried out the subgroup analysis. We found that the interaction was not remarkable after stratification by ethnicity, education level, PIR, marital status, BMI, TC, HDL, HbA1c, BUN, ALT, AST, smoking status, DM, CVD, and hypertension (All *P*_interaction_ > 0.05), revealing that the connection remained stable without affecting by these covariates (Fig. 5). Although the *P* for interaction for subgroups such as alcohol drinking, age, and stroke were all < 0.05, the corresponding 95% CIs of the OR values crossed 1, indicating that these variables did not demonstrate a true interaction with LE8 metrics. In contrast, only the gender subgroup showed a significant interaction, highlighted in red (Fig. 6). Specifically, an OR of 0.96 suggested that, in female participants, higher levels of LE8 were associated with a 4% reduction in the probability of developing PD compared to lower levels (*P*_interaction_ = 0.029). Detailed information was provided in Table S5. This finding suggested that the effect of LE8 on PD was significantly influenced by gender. To further explore the observed gender-specific interactions, we analyzed the general characteristics of female and male participants separately. These analyses provided additional insights into observed gender interactions and their role in modifying the relationship between LE8 metrics and PD. More details were listed in Table S6 and Table S7.

## Discussion

This was the first cross-sectional survey of the association between the CVH status of the US adults via the AHA's updated LE8 metrics and PD. A notably reverse association between LE8 metrics and PD was observed via multivariate logistic regression models, which was consistent with the results of the sensitivity analysis and RCS analysis. Subgroup analysis revealed that gender exerted an effect in modifying this connection, with female exhibiting a stronger connection.

Previous evidence had established that multiple risk factors (such as high fat intake, and DM) contribute to PD etiology and progression [26, 27]. Furthermore, intensive interventions targeting alterable biochemical indices and lifestyles had been confirmed to lead to favorable benefits PD patients [28]. As an improved scoring algorithm that incorporated the sleep health in LS7, the LE8 metrics was validated their practicability and universality in clinical scenarios [8]. A prospective analysis revealed that adhering to a favorable lifestyle and attaining a high LE8 score could prevent 66.8% and 77.3% of severe new-onset nonalcoholic fatty liver disease, which was independent of genetic risks [29]. Unfortunately, the linkage between improved LE8 metrics and PD had never been explored before. Our investigation addressed the knowledge gap in this field, and the logistic regression model suggested that a higher LE8 metrics was significantly associated with a lower risk of developing PD in a dose–response manner. This reverse effect kept consistent with the results of sensitivity analysis and RCS analysis, implying that favorable LE8 metrics might exerted a beneficial role in reducing PD. These findings, along with previous evidence [30], strongly support that the clinicians should shine a spotlight on therapeutic strategies that improve LE8 metrics and reinforce success in pursuing a healthier CVH.

The underlying mechanism linking LE8 metrics and PD remained elusive in the existing literature. Therefore, we could only speculate the potential mechanism based on the current knowledge. Since LE8 metrics integrate by biological and behavioral components, this improved approach underscored that the plausible biological effects might underpin the connection of PD with abnormal metabolic conditions and unhealthy lifestyles, involving crosstalks of some pathways, including inflammation, insulin resistance, oxidative stress, as well as lipid metabolism [6]. For example, inflammation, as a strong trigger of CVD, was conducive to the aggregation of α-Syn and diffusion in the brain, further exacerbating inflammatory responses during PD progression [31, 32]. Besides, a high-fat diet, a pivotal contributor to metabolic disorders in CVD, could increase the vulnerability of the body to neurotoxin injury and dopamine depletion in the substantia nigra, which degenerated the development of PD [33–36]. This study revealed that participants with higher LE8 scores were more likely to have a BMI < 24 kg/m^2^, suggesting that avoiding obesity might be linked to improved CVH. Urgent studies should be conducted to elucidate the precise underpinning mechanism.

This study observed that Non-Hispanic White were the most represented ethnicity, and were positively correlated with LE8 metrics, whereas Non-Hispanic Black were negatively correlated with LE8 metrics. A cautiously conjected factor might be differences in available social resources, health care, combined with the incidence of PD and high LE8 metrics among different ethnic subgroups [37, 38]. Consistent with prior evidence [39], we also concluded the participants with healthier CVH received higher education and held more income than did those with low CVH levels, indirectly supporting our speculation. On the basis of the finding shown here, the sensitivity of LE8 metrics should also be enhanced at the population level in response to policy changes, ethnicity disparities, or other influences. More precise strategies for maintaining a high CVH status would hold great promise for dramatic shrinkage in the future burden PD and improved health equity.

Another interesting finding of this study was that the association between LE8 metrics and PD appeared to be influenced by gender, with female showing a stronger effect compared to male. Gender disparities in PD have been well-documented, with male having twice the probability of developing PD compared to female, as well as differing clinical symptoms and responses to treatment [40]. One possible explanation for our findings was that LE8 metrics themselves might be gender-related, as baseline characteristics indicated that participants with higher LE8 scores were more likely to be female. Additionally, previous studies, such as Fanny et al. [30], reported that higher LE8 metrics had a stronger beneficial effect on reducing the risk of all-cause dementia in female compared to male. Although the joint effect of gender and LE8 metrics on PD remains unclear, these findings highlight the need to investigate potential gender-related mechanism in the LE8-PD relationship. Such mechanism may involve hormonal, genetic, lifestyle, and environmental interactions. Identifying these differences is critical for tailoring interventions to specific populations of PD patients who could benefit most from improving LE8 metrics. In contrast, in other subgroups, such as alcohol drinking and stroke history, the 95% CIs crossed 1, indicating no significant interaction between LE8 metrics and these factors in relation to PD. These results suggested that the effects of LE8 metrics may vary across different subgroups, further emphasizing the importance of investigating population-specific characteristics that could modify the LE8-PD relationship. Future research should prioritize understanding these gender-specific and subgroup-related factors to inform personalized prevention and management strategies for PD and effectively address health disparities.

**Fig. 7:**
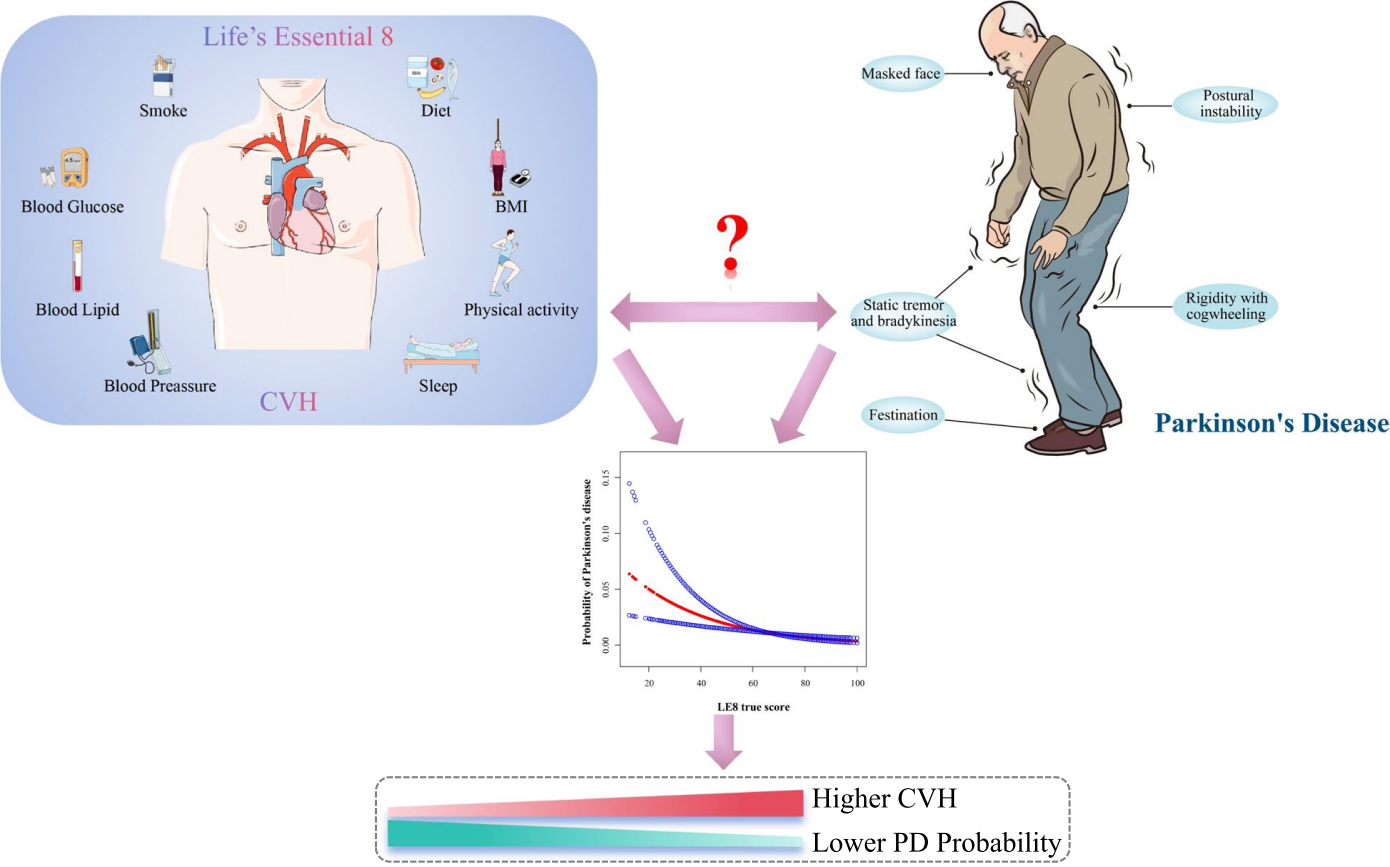
The graphic summary in this study. LE8, Life's essential 8; PD, Parkinson's disease; CVH, cardiovascular health; BMI, body mass index

A graphical summary illustrated the key findings of this study (Fig. 7), showing that pursuing a higher CVH level is associated with a lower probability of developing PD, which has significant implications for clinical practice. LE8 is anticipated to serve as a valuable tool for PD risk assessment, with ongoing efforts to develop a LE8-based nomogram model and explore its integration with environmental factors [41] for improved precision. Since many LE8 components, such as diet, sleep, and physical activity, are modifiable, targeted interventions to address gender-based CVH disparities could potentially help lower the probability of developing PD. Public health initiatives promoting LE8 as a preventive metric could encourage healthier behaviors on a population level, while personalized medicine approaches may use LE8 metrics to tailor lifestyle recommendations for individuals. As highlighted by Nguyen XT et al. [42], the adoption and promotion of lifestyle modifications, with or without direct support from healthcare practitioners, can empower individuals to independently reduce disease burden. If further validated, LE8 holds potential to be integrated into clinical guidelines for PD prevention and management, providing a framework for risk stratification, lifestyle interventions, and public health strategies to reduce the burden of PD. Future studies, particularly large-scale cohort studies or randomized controlled trials, could build upon this study to identify which items form LE8 metrics are most strongly associated with PD and which are less significant. These studies could further validate our findings and provide longitudinal insights. Additionally, exploring the association or causality between LE8 metrics and PD prodromal symptoms [43] will be an exciting and promising research direction.

There were several limitations should be acknowledged. Firstly, the data were based on 7 cycles over a relatively short time period (< 15 years), caution should be exercised when generalizing the findings to secular trends between LE8 metrics and PD as restricted to timeliness. Secondly, we ruled out a comprehensive list of confounding covariates in the analyzes, but those unmeasured or residual confounders should not be neglected. Although we discussed the mechanism underlying the indicated associations between CVH and PD, the specific mechanism of this effect needs to be further explored at the molecular or cellular level. And PD identification in this study was based on the use of specific medications commonly prescribed for its treatment. While this approach carried an inherent risk of bias that might impact result validity, it is worth noting that even clinical PD diagnoses remained speculative, as a definitive diagnosis could only be confirmed through post-mortem examination [44, 45]. Notably, the cross-sectional nature of the NHANES study inherently limited the ability to establish causal inferences between LE8 metrics and PD, as it prevented the determination of temporal relationships or directionality. To establish robust causality, future research should prioritize preclinical and clinical randomized controlled trials with large sample sizes, involving not only US citizens but also populations from other countries.

## Conclusions

Taken together, this study hinted at a reverse association between LE8 metrics and PD in a dose–response pattern using NHANES 2005–2018 data, more pronounced in female compared to male. These findings highlight the potential of the LE8 metrics to guide targeted strategies for addressing gender-based CVH disparities, offering beneficial insights for the tertiary prevention of PD, reduction of health expenditure, and extension of healthy longevity, with the promise of greater social well-being and health equity. The key for future investigations is to clarify their robust causal linkage in large-scale and multiregional randomized controlled trials and further explore the underpinning mechanism of this association.

## Supplementary Information

Supplementary Material 1.

Supplementary Material 2.

## Data Availability

All NHANES data supporting the findings of this study can be found here https://wwwn.cdc.gov/nchs/nhanes/default.aspx, which are available for public use. And they are also obtainable from the corresponding author, upon reasonable request.
