# Supplementary material for: Association between life's essential 8 and Parkinson's disease: a case–control study
Source: BMC Public Health. 2025 Feb 1;25:411. doi: 10.1186/s12889-025-21648-0 (PMC11786534; doi:10.1186/s12889-025-21648-0)
Supplement: Supplementary file 1 — Supplementary Material 1. [file 12889_2025_21648_MOESM1_ESM.docx]

**Table S1** The overview of missing values of each variable.

| **Covariates** | **N** | **Missing N** | **Missing rate (%)** |
| --- | --- | --- | --- |
| Age | 26,975 | 0 | 0.00 |
| Gender | 26,975 | 0 | 0.00 |
| Ethnicity | 26,975 | 0 | 0.00 |
| Education level | 26,956 | 19 | 0.07 |
| PIR | 24,889 | 2086 | 7.73 |
| Marital status | 26,967 | 8 | 0.03 |
| PA | 18,468 | 8,507 | 31.54 |
| BMI | 26,975 | 0 | 0.00 |
| TC | 26,975 | 0 | 0.00 |
| HDL | 26,975 | 0 | 0.00 |
| HbA1c | 26,955 | 20 | 0.07 |
| BUN | 26879 | 96 | 0.36 |
| ALT | 26,814 | 161 | 0.60 |
| AST | 26,801 | 174 | 0.65 |
| Alcohol drinking | 25,155 | 1,820 | 6.75 |
| Smoking status | 26,975 | 0 | 0.00 |
| CVD | 26,973 | 2 | 0.01 |
| DM | 26,975 | 0 | 0.00 |
| Hypertension | 26,975 | 0 | 0.00 |
| Stroke | 26,945 | 30 | 0.11 |

Note: PIR, poverty-to-income ratio; BMI, body mass index; PA, physical activity; TC, total cholesterol; HDL, high-density lipoprotein; HbA1c, glycated hemoglobin; BUN, blood urea nitrogen; ALT, alanine transaminase; AST, aspartate aminotransferase; CVD, cardiovascular diseases; DM, diabetes mellitus. Missing rate (%) = Missing N/Total N × 100%.

**Table S2** Missing data distribution by LE8 tertile grouping.

| **Variables** | **Low** | **Moderate** | **High** |
| --- | --- | --- | --- |
| Gender | 0 | 0 | 0 |
| Age | 0 | 0 | 0 |
| Ethnicity | 0 | 0 | 0 |
| Education level | 8 | 8 | 3 |
| Marital status | 4 | 2 | 2 |
| PIR | 691 | 696 | 699 |
| BMI | 0 | 0 | 0 |
| Stroke | 17 | 5 | 8 |
| CVD | 2 | 0 | 0 |
| Smoking status | 0 | 0 | 0 |
| DM | 0 | 0 | 0 |
| Hypertension | 0 | 0 | 0 |
| HbA1c | 3 | 5 | 12 |
| ALT | 60 | 61 | 40 |
| AST | 63 | 65 | 46 |
| BUN | 37 | 32 | 27 |
| TC | 0 | 0 | 0 |
| HDL | 0 | 0 | 0 |
| Alcohol drinking | 690 | 572 | 558 |
| PA | 5058 | 2403 | 1046 |

Note: PIR, poverty-to-income ratio; BMI, body mass index; PA, physical activity; TC, total cholesterol; HDL, high-density lipoprotein; HbA1c, glycated hemoglobin; BUN, blood urea nitrogen; ALT, alanine transaminase; AST, aspartate aminotransferase; CVD, cardiovascular diseases; DM, diabetes mellitus.

**Table S3** The descriptive statistics of each variable before and after multiple imputations.

| **Variables** | **Before Multiple Imputation** | | | | **After Multiple Imputation** | | | |
| --- | --- | --- | --- | --- | --- | --- | --- | --- |
|  | **Mean** | **Median** | **Q1** | **Q3** | **Mean** | **Median** | **Q1** | **Q3** |
| Gender | 1.49 | 1 | 1 | 2 | 1.49 | 1 | 1 | 2 |
| Age | 49.97 | 50 | 35 | 64 | 49.97 | 50 | 35 | 64 |
| Ethnicity | 2.69 | 3 | 2 | 3 | 2.69 | 3 | 2 | 3 |
| Education level | 1.45 | 2 | 1 | 2 | 1.45 | 2 | 1 | 2 |
| Marital status | 0.61 | 1 | 0 | 1 | 0.61 | 1 | 0 | 1 |
| PIR | 2.59 | 2.20 | 1.16 | 4.19 | 2.58 | 2.20 | 1.16 | 4.17 |
| BMI | 29.18 | 28.10 | 24.42 | 32.60 | 29.18 | 28.10 | 24.42 | 32.60 |
| Stroke | 0.04 | 0 | 0 | 0 | 0.04 | 0 | 0 | 0 |
| CVD | 0.11 | 0 | 0 | 0 | 0.11 | 0 | 0 | 0 |
| Smoking status | 0.65 | 0 | 0 | 1 | 0.65 | 0 | 0 | 1 |
| DM | 0.14 | 0 | 0 | 0 | 0.14 | 0 | 0 | 0 |
| Hypertension | 0.43 | 0 | 0 | 1 | 0.43 | 0 | 0 | 1 |
| HbA1c | 5.70 | 5.5 | 5.2 | 5.8 | 5.70 | 5.5 | 5.2 | 5.8 |
| ALT | 24.85 | 20 | 16 | 28 | 24.85 | 20 | 16 | 28 |
| AST | 25.34 | 25.34 | 19 | 27 | 25.35 | 23 | 19 | 27 |
| BUN | 13.79 | 13.79 | 10 | 16 | 13.79 | 13 | 10 | 16 |
| TC | 192.90 | 190 | 164 | 218 | 192.90 | 190 | 164 | 218 |
| HDL | 53.14 | 51 | 42 | 62 | 53.14 | 51 | 42 | 62 |
| Alcohol drinking | 1.90 | 2 | 1 | 3 | 1.90 | 2 | 1 | 3 |
| PA | 4559.88 | 1920 | 720 | 5520 | 4305.56 | 1920 | 720 | 5040 |
| LE8 | 66.42 | 66.88 | 56.25 | 76.88 | 66.42 | 66.88 | 56.25 | 76.88 |
| PD | 0.01 | 0 | 0 | 0 | 0.01005 | 0 | 0 | 0 |

Note: PIR, poverty-to-income ratio; BMI, body mass index; TC, total cholesterol; HDL, high-density lipoprotein; HbA1c, glycated hemoglobin; BUN, blood urea nitrogen; ALT, alanine transaminase; AST, aspartate aminotransferase; CVD, cardiovascular diseases; DM, diabetes mellitus; PA, physical activity; LE8, life's essential 8; PD, Parkinson's disease.

**Table S4** The general characteristics of participants with and without physical activity data, weighted.

| **Characteristics** | **Total eligible participants** | | ***P*** |
| --- | --- | --- | --- |
|  | **With PA** | **Without PA** |  |
| **N** | 18,468 | 8,507 |  |
| **Age**, years | 51.89±0.38 | 46.45±0.26 | <0.001 |
| **Ethnicity** |  |  | 0.015 |
| Mexican American | 8.58±0.79 | 7.54±0.63 |  |
| Non-Hispanic Black | 11.27±0.87 | 9.74±0.64 |  |
| Non-Hispanic White | 68.79±1.52 | 70.53±1.27 |  |
| Other Race | 11.36±0.75 | 12.19±0.58 |  |
| **Education level** |  |  | <0.001 |
| <High School | 7.33±0.50 | 3.51±0.20 |  |
| High School | 38.62±0.81 | 30.68±0.90 |  |
| >High School | 54.05±0.91 | 65.81±0.99 |  |
| **PIR** |  |  | <0.001 |
| <1.3 | 21.58±0.73 | 18.56±0.65 |  |
| 1.3≤PIR<3.5 | 38.82±0.93 | 34.73±0.77 |  |
| ≥3.5 | 39.60±1.22 | 46.71±1.08 |  |
| **Marital status** |  |  | 0.470 |
| Unpartnered | 34.63±0.80 | 35.29±0.72 |  |
| Partnered | 65.37±0.80 | 64.71±0.72 |  |
| **BMI**, kg/m^2^ |  |  | <0.001 |
| <24 | 19.38±0.59 | 24.82±0.56 |  |
| ≥24 | 80.62±0.59 | 75.18±0.56 |  |
| **TC**, mg/dL | 195.00±0.57 | 193.82±0.58 | 0.119 |
| **HDL**, mg/dL | 52.90±0.27 | 54.04±0.23 | 0.001 |
| **HbA1c**, % | 5.68±0.02 | 5.53±0.01 | <0.001 |
| **BUN**, mg/dL | 14.01±0.11 | 13.56±0.08 | <0.001 |
| **ALT**, U/L | 24.80±0.28 | 25.16±0.16 | 0.235 |
| **AST**, U/L | 25.16±0.22 | 25.18±0.13 | 0.949 |
| **LE8** | 58.47±0.31 | 72.14±0.22 | <0.001 |
| **LE8 tertiles** |  |  | <0.001 |
| 0<LE8≤60.0 | 55.17±1.01 | 18.29±0.50 |  |
| 60.0<LE8≤73.0 | 29.49±0.77 | 32.60±0.56 |  |
| 73.0<LE8≤100.0 | 15.34±0.69 | 49.11±0.78 |  |
| **Alcohol drinking** |  |  | <0.001 |
| None | 13.08±0.66 | 9.34±0.50 |  |
| Former | 35.43±0.74 | 39.33±0.71 |  |
| Light | 15.39±0.59 | 18.84±0.41 |  |
| Moderate | 17.16±0.64 | 21.12±0.55 |  |
| Heavy | 18.94±0.63 | 11.38±0.40 |  |
| **Smoking status** |  |  | <0.001 |
| Never | 51.98±0.83 | 56.42±0.72 |  |
| Former | 26.86±0.74 | 25.26±0.57 |  |
| Current | 21.16±0.70 | 18.33±0.48 |  |
| **CVD** |  |  | <0.001 |
| No | 86.46±0.54 | 93.25±0.29 |  |
| Yes | 13.54±0.54 | 6.75±0.29 |  |
| **DM** |  |  | <0.001 |
| No | 84.73±0.54 | 91.25±0.28 |  |
| Yes | 15.27±0.54 | 8.75±0.28 |  |
| **Hypertension** |  |  | <0.001 |
| No | 53.54±0.77 | 65.63±0.62 |  |
| Yes | 46.46±0.77 | 34.37±0.62 |  |
| **Stroke** |  |  | <0.001 |
| No | 95.07±0.30 | 98.12±0.13 |  |
| Yes | 13.54±0.30 | 6.75±0.13 |  |
| **PD** |  |  | 0.013 |
| No | 98.75±0.16 | 99.15±0.09 |  |
| Yes | 1.25±0.16 | 0.85±0.09 |  |

Note: The continuous variables were described by weighted mean ± standard error (SE), while the categorical variables were described by weighted percentage (%) ± SE. The weighted ANOVA analysis was used to assess the differences of the continuous variables, while the weighted chi-square test was used to assess the differences of the categorical variables. PIR, poverty-to-income ratio; BMI, body mass index; TC, total cholesterol; HDL, high-density lipoprotein; HbA1c, glycated hemoglobin; BUN, blood urea nitrogen; ALT, alanine transaminase; AST, aspartate aminotransferase; CVD, cardiovascular diseases; DM, diabetes mellitus; PA, physical activity; LE8, life's essential 8; PD, Parkinson's disease.

**Table S5** Subgroup analysis and interaction test of association between LE8 metrics and PD.

| **Subgroup name** | **OR (95% CI) *P*** | | ***P* for interaction** |
| --- | --- | --- | --- |
| **Age**, years |  |  | <0.001 |
| <65 | 0.96 (0.95-0.97) | <0.001 |  |
| ≥65 | 0.99 (0.98-1.00) | 0.069 |  |
| **Gender** |  |  | 0.029 |
| Female | 0.96 (0.95-0.97) | <0.001 |  |
| Male | 0.98 (0.97-0.99) | <0.001 |  |
| **Ethnicity** |  |  | 0.532 |
| Mexican American | 0.96 (0.93-0.99) | 0.011 |  |
| Non-Hispanic Black | 0.98 (0.95-1.00) | 0.022 |  |
| Non-Hispanic White | 0.97 (0.96-0.98) | <0.001 |  |
| Other Race | 0.95 (0.93-0.98) | <0.001 |  |
| **Education level** |  |  | 0.322 |
| <High School | 0.95 (0.92-0.98) | <0.001 |  |
| High School | 0.97 (0.96-0.98) | <0.001 |  |
| >High School | 0.97 (0.96-0.98) | <0.001 |  |
| **PIR** |  |  | 0.813 |
| <1.3 | 0.97 (0.95-0.98) | <0.001 |  |
| 1.3≤PIR<3.5 | 0.97 (0.96-0.98) | <0.001 |  |
| ≥3.5 | 0.97 (0.95-0.98) | <0.001 |  |
| **Marital status** |  |  | 0.427 |
| Unpartnered | 0.96 (0.95-0.98) | <0.001 |  |
| Partnered | 0.97 (0.96-0.98) | <0.001 |  |
| **BMI**, kg/m^2^ |  |  | 0.093 |
| <24 | 0.98 (0.96-1.00) | 0.024 |  |
| ≥24 | 0.96 (0.95-0.97) | <0.001 |  |
| **TC**, mg/dL |  |  | 0.225 |
| ≤200 | 0.96 (0.95-0.97) | <0.001 |  |
| >200 | 0.97 (0.96-0.99) | <0.001 |  |
| **HDL**, mg/dL |  |  | 0.638 |
| ≤60 | 0.96 (0.95-0.97) | <0.001 |  |
| >60 | 0.97 (0.95-0.98) | <0.001 |  |
| **HbA1c**, % |  |  | 0.425 |
| ≤6 | 0.97 (0.96-0.98) | <0.001 |  |
| >6 | 0.96 (0.94-0.98) | <0.001 |  |
| **BUN**, mg/dL |  |  | 0.693 |
| ≤12 | 0.97 (0.95-0.98) | <0.001 |  |
| >12 | 0.97 (0.96-0.98) | <0.001 |  |
| **ALT**, U/L |  |  | 0.062 |
| ≤40 | 0.97 (0.96-0.98) | <0.001 |  |
| >40 | 0.94 (0.91-0.97) | <0.001 |  |
| **AST**, U/L |  |  | 0.480 |
| ≤40 | 0.97 (0.96-0.98) | <0.001 |  |
| >40 | 0.96 (0.92-0.99) | 0.012 |  |
| **Alcohol drinking** |  |  | 0.012 |
| None | 0.97 (0.95-0.99) | 0.014 |  |
| Former | 0.97 (0.95-0.98) | <0.001 |  |
| Light | 0.95 (0.93-0.98) | <0.001 |  |
| Moderate | 0.95 (0.93-0.98) | <0.001 |  |
| Heavy | 0.99 (0.98-1.01) | 0.457 |  |
| **Smoking status** |  |  | 0.967 |
| Never | 0.97 (0.95-0.98) | <0.001 |  |
| Former | 0.96 (0.95-0.98) | <0.001 |  |
| Current | 0.96 (0.95-0.98) | <0.001 |  |
| **CVD** |  |  | 0.066 |
| No | 0.97 (0.96-0.98) | <0.001 |  |
| Yes | 0.99 (0.97-1.00) | 0.099 |  |
| **DM** |  |  | 0.965 |
| No | 0.97 (0.96-0.98) | <0.001 |  |
| Yes | 0.97 (0.95-0.99) | 0.001 |  |
| **Hypertension** |  |  | 0.647 |
| No | 0.97 (0.96-0.98) | <0.001 |  |
| Yes | 0.98(0.97-0.99) | <0.001 |  |
| **Stroke** |  |  | 0.023 |
| No | 0.97 (0.96-0.98) | <0.001 |  |
| Yes | 1.00 (0.97-1.02) | 0.821 |  |

Note: PIR, poverty-to-income ratio; BMI, body mass index; TC, total cholesterol; HDL, high-density lipoprotein; HbA1c, glycated hemoglobin; BUN, blood urea nitrogen; ALT, alanine transaminase; AST, aspartate aminotransferase; CVD, cardiovascular diseases; DM, diabetes mellitus; OR, odds ratio; 95%CI, 95% confidence interval.

**Table S6** General characteristics of the female participants in this study, weighted.

| **Characteristics**  **Female** | **LE8 metrics** | | | ***P*** |
| --- | --- | --- | --- | --- |
|  | **Low**  **(0<LE8≤60.0)** | **Moderate**  **(60.0<LE8≤73.0)** | **High**  **(73.0<LE8≤100.0)** |  |
| **N** | 4,514 | 4,585 | 4,742 |  |
| **Age**, years | 54.52 ±0.24 | 50.18±0.30 | 43.47±0.34 | <0.001 |
| **Ethnicity** |  |  |  | <0.001 |
| Mexican American | 6.40±0.67 | 7.81±0.67 | 7.57±0.68 |  |
| Non-Hispanic Black | 15.32±1.06 | 12.07±0.95 | 6.80±0.53 |  |
| Non-Hispanic White | 68.50±1.63 | 68.62±1.55 | 71.64±1.30 |  |
| Other Race | 9.77±0.669 | 11.51±0.67 | 13.99±0.85 |  |
| **Education level** |  |  |  | <0.001 |
| <High School | 6.60±0.52 | 4.50±0.33 | 2.63±0.25 |  |
| High School | 46.84±1.15 | 34.56±0.90 | 19.29±0.90 |  |
| >High School | 46.55±1.22 | 60.94±0.95 | 78.08±0.96 |  |
| **PIR** |  |  |  | <0.001 |
| <1.3 | 30.08±1.07 | 21.29±0.79 | 14.60±0.67 |  |
| 1.3≤PIR<3.5 | 42.27±1.10 | 37.37±1.03 | 31.37±1.08 |  |
| ≥3.5 | 27.65±1.34 | 41.34±1.26 | 54.03±1.37 |  |
| **Marital status** |  |  |  | <0.001 |
| Unpartnered | 45.71±0.98 | 39.14±1.05 | 34.00±1.05 |  |
| Partnered | 54.29±0.98 | 60.86±1.05 | 66.00±1.05 |  |
| **BMI**, kg/m^2^ |  |  |  | <0.001 |
| <24 | 8.48±0.65 | 20.23±0.91 | 46.98 ±1.04 |  |
| ≥24 | 91.52±0.65 | 79.77±0.91 | 53.02±1.04 |  |
| **PA**, MET-h/week | 3175.65±169.94 | 3489.66±120.13 | 3344.43±97.90 | 0.174 |
| **TC**, mg/dL | 208.20±0.69 | 200.52±0.75 | 187.03±0.51 | <0.001 |
| **HDL**, mg/dL | 52.48±0.21 | 58.05±0.23 | 64.39±0.28 | <0.001 |
| **HbA1c**, % | 5.99±0.02 | 5.53±0.01 | 5.27±0.01 | <0.001 |
| **BUN**, mg/dL | 13.79±0.09 | 13.15±0.10 | 12.40±0.09 | <0.001 |
| **ALT**, U/L | 22.87±0.33 | 21.09±0.23 | 19.43±0.19 | <0.001 |
| **AST**, U/L | 24.25±0.23 | 23.16±0.17 | 22.83±0.18 | 0.001 |
| **Alcohol drinking** |  |  |  | <0.001 |
| None | 14.60±0.74 | 14.83±0.71 | 13.51±0.86 |  |
| Former | 21.75±0.95 | 13.31±0.73 | 7.76±0.45 |  |
| Light | 28.70±1.09 | 31.95±1.08 | 36.26±1.02 |  |
| Moderate | 18.07±0.79 | 21.78±0.91 | 26.95±0.90 |  |
| Heavy | 16.88±0.77 | 18.12±0.84 | 15.52±0.68 |  |
| **Smoking status** |  |  |  | <0.001 |
| Never | 40.45±0.85 | 60.50±0.88 | 77.40±0.96 |  |
| Former | 25.20±0.98 | 21.74±0.91 | 18.41±0.81 |  |
| Current | 34.35±0.88 | 17.76±0.75 | 4.19±0.40 |  |
| **CVD** |  |  |  | <0.001 |
| No | 84.44±0.69 | 93.17±0.38 | 97.71±0.28 |  |
| Yes | 15.56±0.69 | 6.83±0.38 | 2.29±0.28 |  |
| **DM** |  |  |  | <0.001 |
| No | 75.94±0.77 | 91.32±0.54 | 98.70±0.20 |  |
| Yes | 24.06±0.77 | 8.68±054 | 1.30±0.20 |  |
| **Hypertension** |  |  |  | <0.001 |
| No | 36.31±0.92 | 59.32±0.84 | 84.98±0.70 |  |
| Yes | 63.69±0.92 | 40.68±0.84 | 15.02±0.70 |  |
| **Stroke** |  |  |  | <0.001 |
| No | 93.56±0.46 | 97.29±0.25 | 99.14±0.18 |  |
| Yes | 6.44±0.46 | 2.71±0.25 | 0.86±0.18 |  |
| **PD** |  |  |  | <0.001 |
| No | 97.87±0.26 | 98.84±0.21 | 99.42±0.15 |  |
| Yes | 2.13±0.26 | 1.16±0.21 | 0.58±0.15 |  |

Note: The continuous variables were described by weighted mean ± standard error (SE), while the categorical variables were described by weighted percentage (%) ± SE. The weighted ANOVA analysis was used to assess the differences of the continuous variables, while the weighted chi-square test was used to assess the differences of the categorical variables.

PIR, poverty-to-income ratio; BMI, body mass index; TC, total cholesterol; HDL, high-density lipoprotein; HbA1c, glycated hemoglobin; BUN, blood urea nitrogen; ALT, alanine transaminase; AST, aspartate aminotransferase; CVD, cardiovascular diseases; DM, diabetes mellitus; PA, physical activity; LE8, life's essential 8; PD, Parkinson's disease.

**Table S7** General characteristics of the male participants in this study, weighted.

| **Characteristics**  **Male** | **LE8 metrics** | | | ***P*** |
| --- | --- | --- | --- | --- |
|  | **Low**  **(0<LE8≤60.0)** | **Moderate**  **(60.0<LE8≤73.0)** | **High**  **(73.0<LE8≤100.0)** |  |
| **N** | 4,244 | 4,317 | 4,573 |  |
| **Age**, years | 51.35±0.33 | 47.75±0.33 | 43.78±0.40 | <0.001 |
| **Ethnicity** |  |  |  | <0.001 |
| Mexican American | 8.72±0.90 | 8.96±0.80 | 7.67±0.63 |  |
| Non-Hispanic Black | 12.38±0.92 | 9.37±0.58 | 7.29±0.53 |  |
| Non-Hispanic White | 68.34±1.55 | 70.82±1.31 | 71.28±1.36 |  |
| Other Race | 10.55±0.68 | 10.85±0.66 | 13.77±0.85 |  |
| **Education level** |  |  |  | <0.001 |
| <High School | 7.32±0.53 | 4.53±0.33 | 3.42±0.32 |  |
| High School | 44.2±1.13 | 36.28±1.17 | 24.78±1.13 |  |
| >High School | 48.49±1.28 | 59.19±1.24 | 71.8±1.20 |  |
| **PIR** |  |  |  | <0.001 |
| <1.3 | 22.43±1.03 | 17.80±0.74 | 14.08±0.72 |  |
| 1.3≤PIR<3.5 | 39.97±1.11 | 35.98±1.16 | 31.70±1.12 |  |
| ≥3.5 | 37.61±1.27 | 46.23±1.29 | 54.23±1.30 |  |
| **Marital status** |  |  |  | 0.746 |
| Unpartnered | 30.31±1.01 | 31.08±1.07 | 31.31±1.01 |  |
| Partnered | 69.69±1.01 | 68.92±1.07 | 68.69±1.01 |  |
| **BMI**, kg/m^2^ |  |  |  | <0.001 |
| <24 | 7.84±0.59 | 13.91±0.65 | 30.58±1.01 |  |
| ≥24 | 92.16±0.59 | 86.09±0.65 | 69.42±1.01 |  |
| **PA**, MET-h/week | 5568.07±266.86 | 5908.04±184.08 | 5433.18±156.79 | 0.067 |
| **TC**, mg/dL | 202.8±0.94 | 193.15±0.97 | 180.08±0.71 | <0.001 |
| **HDL**, mg/dL | 43.81±0.25 | 47.3±0.28 | 51.69±0.32 | <0.001 |
| **HbA1c**, % | 6.03±0.02 | 5.56±0.02 | 5.32±0.01 | <0.001 |
| **BUN**, mg/dL | 14.54±0.13 | 14.23±0.12 | 14.44±0.10 | 0.195 |
| **ALT**, U/L | 32.3±0.52 | 30.3±0.38 | 26.85±0.30 | <0.001 |
| **AST**, U/L | 28.05±0.37 | 27.05±0.24 | 26.6±0.28 | 0.006 |
| **Alcohol drinking** |  |  |  | <0.001 |
| None | 4.24±0.39 | 6.12±0.57 | 7.96±0.77 |  |
| Former | 36.97±1.10 | 41.18±1.14 | 51.17±1.22 |  |
| Light | 11.96±0.81 | 13.73±0.77 | 12.22±0.57 |  |
| Moderate | 26.99±0.98 | 26.10±1.15 | 18.78±0.82 |  |
| Heavy | 19.85±0.99 | 12.88±0.69 | 9.87±0.58 |  |
| **Smoking status** |  |  |  | <0.001 |
| Never | 25.43±1.03 | 43.98±0.99 | 68.1±1.02 |  |
| Former | 34.28±0.87 | 32.5±0.92 | 25.78±0.92 |  |
| Current | 40.3±1.00 | 23.52±0.78 | 6.12±0.51 |  |
| **CVD** |  |  |  | <0.001 |
| No | 82.70±0.81 | 90.55±0.53 | 94.77±0.41 |  |
| Yes | 17.30±0.81 | 9.45±0.53 | 5.23±0.41 |  |
| **DM** |  |  |  | <0.001 |
| No | 75.39±0.86 | 90.52±0.60 | 96.93±0.32 |  |
| Yes | 24.61±0.86 | 9.48±0.60 | 3.07±0.32 |  |
| **Hypertension** |  |  |  | <0.001 |
| No | 40.47±1.02 | 59.00±1.07 | 78.08±0.81 |  |
| Yes | 59.53±1.02 | 41.00±1.07 | 21.92±0.81 |  |
| **Stroke** |  |  |  | <0.001 |
| No | 95.07±0.40 | 98.31±0.19 | 98.68±0.20 |  |
| Yes | 4.93±0.40 | 1.69±0.19 | 1.32±0.20 |  |
| **PD** |  |  |  | 0.094 |
| No | 98.99±0.21 | 99.41±0.14 | 99.42±0.14 |  |
| Yes | 1.01±0.21 | 0.59±0.14 | 0.58±0.14 |  |

Note: The continuous variables were described by weighted mean ± standard error (SE), while the categorical variables were described by weighted percentage (%) ± SE. The weighted ANOVA analysis was used to assess the differences of the continuous variables, while the weighted chi-square test was used to assess the differences of the categorical variables.

PIR, poverty-to-income ratio; BMI, body mass index; TC, total cholesterol; HDL, high-density lipoprotein; HbA1c, glycated hemoglobin; BUN, blood urea nitrogen; ALT, alanine transaminase; AST, aspartate aminotransferase; CVD, cardiovascular diseases; DM, diabetes mellitus; PA, physical activity; LE8, life's essential 8; PD, Parkinson's disease.

**Figure S1 The missing values details of each variable.** The overview of missing values of each variable (**A**). The missing values distribution by LE8 tertile grouping (**B**). PIR, poverty-to-income ratio; BMI, body mass index; TC, total cholesterol; HDL, high-density lipoprotein; HbA1c, glycated hemoglobin; BUN, blood urea nitrogen; ALT, alanine transaminase; AST, aspartate aminotransferase; CVD, cardiovascular diseases; DM, diabetes mellitus; PA, physical activity; LE8, life's essential 8; PD, Parkinson's disease.
