# Supplementary figures and images for: Association between life's essential 8 and Parkinson's disease: a case–control study
Source: BMC Public Health. 2025 Feb 1;25:411. doi: 10.1186/s12889-025-21648-0 (PMC11786534; doi:10.1186/s12889-025-21648-0)

A

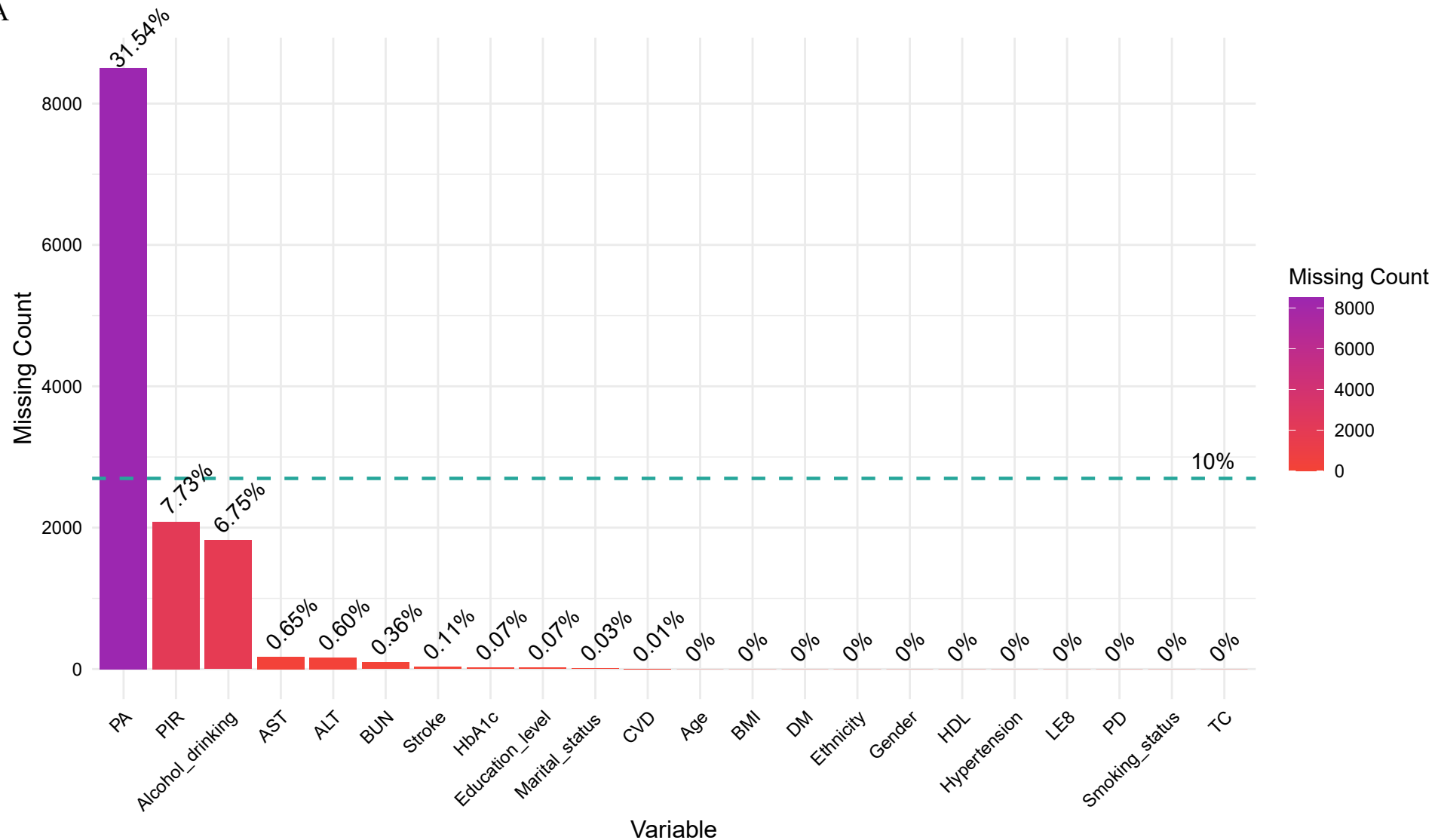

B

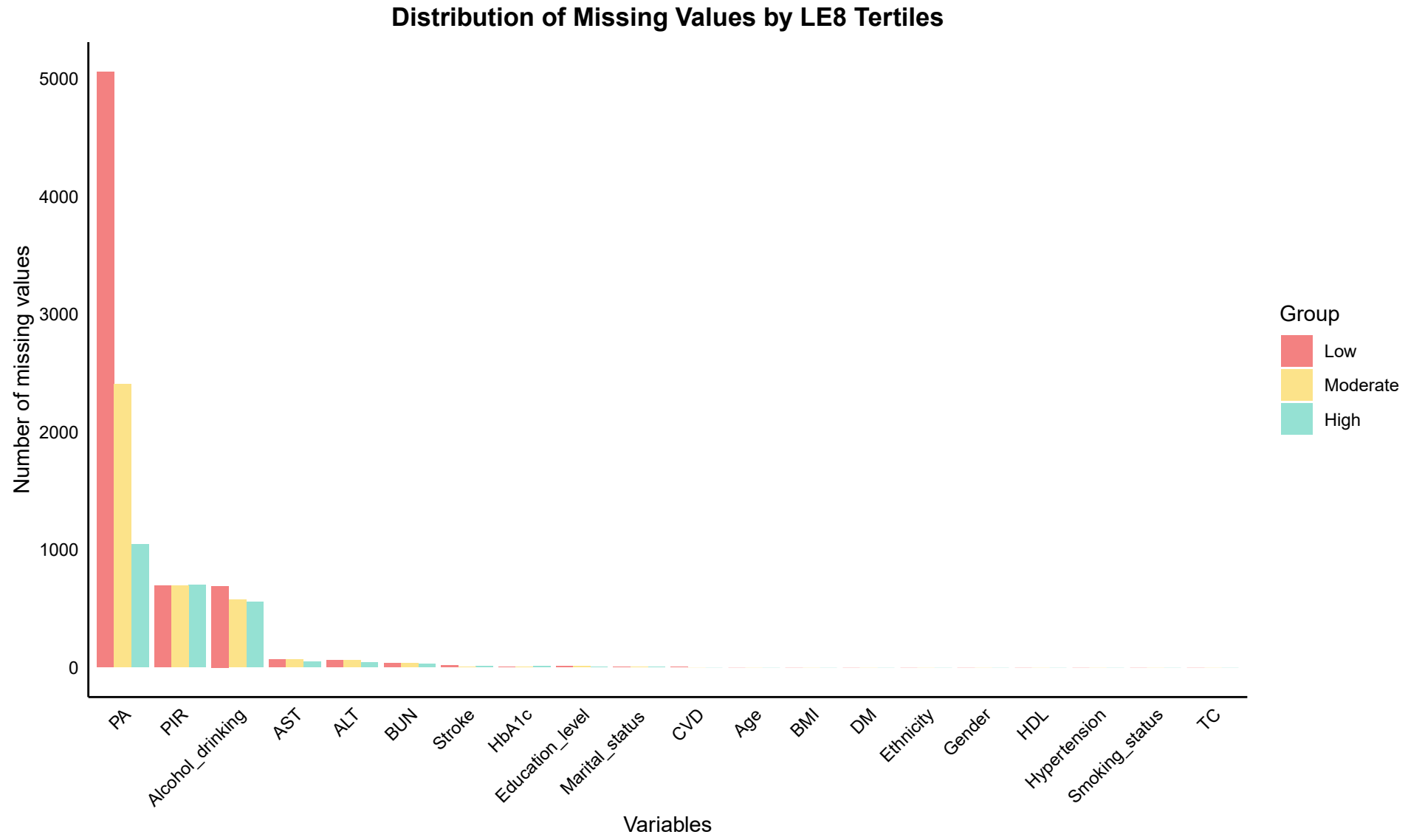

Supplement: Supplementary file 2 — Supplementary Material 2. [file 12889_2025_21648_MOESM2_ESM.pdf]
